# Prevalence, patterns, and perceived value of complementary and alternative medicine among cancer patients: a cross-sectional, descriptive study

**DOI:** 10.1186/s12906-017-1853-6

**Published:** 2017-06-30

**Authors:** Mandreker Bahall

**Affiliations:** 1 School of Medicine and Arthur Lok Jack Graduate School of Business, University of the West Indies, St. Augustine, Trinidad and Tobago; 2 Department of Medicine, San Fernando General Hospital, Chancery Lane, San Fernando, Trinidad and Tobago; 3 House #57 LP 62, Calcutta Road Number 3, McBean, Couva, Trinidad Trinidad and Tobago

**Keywords:** Cancer treatment, Complementary and alternative medicine, Non-disclosure, Patient satisfaction, Side-effect

## Abstract

**Background:**

Sophisticated conventional medicine (CM) has brought significant advances to cancer prevention, detection, and treatment. However, many cancer patients still turn to complementary and alternative medicine (CAM) treatment. This study explored the prevalence, patterns, and perceived value of CAM among cancer patients.

**Methods:**

This quantitative descriptive study was conducted between March 1, 2015, and July 31, 2015, among a cross-sectional, convenience sample of patients from the Oncology Department of San Fernando General Hospital in Trinidad and Tobago. Face-to-face interviews were conducted at the oncology clinic and treatment suite after obtaining informed consent. Data analysis included descriptive analysis, chi-square tests, and binary logistic regression analysis.

**Results:**

The prevalence of CAM use among a sample of 350 cancer patients was 39.1% (39.6% for breast cancer, 44.4% for prostate cancer, 37% for ovarian cancer, and 38.7% for colon cancer patients). Herbs were the most common type of CAM used (93.4%), followed by spiritual therapy (73.7%). CAM use was more prevalent among females (68.6%), Indo-Trinidadians (63.5%), and patients aged 41–50 years (37.2%). The majority (70%–80%) rated CAM efficacy on perceived value. CAM was used mainly because of a desire to try anything that might help (67.6%), followed by it being congruent with the patients’ beliefs (59.1%). Patients knew about CAM mainly through friends (69.3%) and family (69.3%). Most patients were generally satisfied (93.6%) and considered CAM helpful (89.8%), but the majority never informed their health care provider of CAM use (78.8%). Patients reported the simultaneous use of more than one type of CAM, without considering or knowing of possible side-effects. The perceived value of CAM included empowerment, control, cure, and improved quality of life. CAM use was associated with age, but no predictors of CAM use could be identified.

**Conclusion:**

Medicinal herbs and spiritual therapy are commonly used among cancer patients because of perceived benefits and satisfaction. CAM use is more prevalent among females, Indo-Trinidadians, and patients aged 41–50 years old. There are no useful predictors of CAM use. More than one type of CAM is commonly used simultaneously without disclosure to health care providers.

## Background

Complementary and alternative medicine (CAM) is defined as “a group of diverse medical and health care systems, practices, and products that are not generally considered part of conventional medicine” [1]. It includes herbs, spiritual therapies (prayers, faith healing, divinations, meditation, psychic therapy, folk magic/sorcery [*obeah*], and mind–body techniques), dietary supplements, biofeedback, hypnosis, acupuncture, Ayurveda, homeopathy, naturopathy, Chinese medicine, chiropractic, massage, *tai chi*, yoga, electromagnetic therapy, kinesiology, *reiki*, and *qigong*. According to the WHO, “the term complementary and alternative medicine is used in some countries to refer to a broad set of health care practices that are not part of the country's own tradition and are not integrated into the dominant health care system” [2]. The overall prevalence of CAM was reported as being 36% in the US (2007), 26% in the UK (2005), and 52% in Australia (2004) [3]. The global prevalence is reported to be 9.8%–76.0% [3].

In Trinidad and Tobago, cancer is one of the top 10 causes of death [4]. Treatment with conventional medicine (CM) has caused significant advances in the prevention, detection, and treatment of cancer [5]. Nonetheless, many patients choose CAM over CM in the hope of maintaining wellness and curing the disease [6–9]. The decision to use CAM is typically influenced by factors such as poor doctor–patient communication, the emotional effect of a cancer diagnosis, perceived severity of conventional treatment side-effects, the individual’s need for decision-making control, and strong beliefs in holistic healing and the mind–body–spirit connection [10]. CM focuses on curative aspects without focusing on the social, psychological, and spiritual needs of the patient [11]. CAM therefore fills this void. Despite the perceived benefits and influences, only a small number of patients refuse CM and prefer CAM alone [9]. These numbers are increasing steadily, with patients reporting continued perceived efficacy since the early 1980s [9, 12–20].

With respect to cancer patients, a European study comprising 956 patients, conducted in 14 countries, revealed that the prevalence of CAM varies markedly among patients with different types of cancer: colon cancer (32.7%), breast cancer (44.7%), lung cancer (23.6%), pancreatic cancer (56.3%), brain cancer (50%), head and neck cancer (22.7%) [21]. A Canadian study revealed CAM usage to be 29.8% among men diagnosed with prostate cancer [22]. The types of CAM used also vary according to the types of cancer: vitamin E, saw palmetto, and selenium are used for prostatic cancer [22, 23]; vitamin A, selenium, phytoestrogens, and traditional Chinese medicine (coumarin, flavonoids) for breast cancer; psychological and spiritual therapies for colorectal cancer [24]; vitamins and minerals for ovarian cancer [25]; herbal plants for lung cancer; and cyclopamine (a steroidal alkaloid extracted from *Veratrum californicum*) for pancreatic cancer. Spiritual therapy is also widely implemented by cancer patients [26].

However, the CAM use and practices among cancer patients in Trinidad and Tobago are unknown. This study therefore explored the prevalence, patterns, and perceived value of CAM among adult cancer patients in Trinidad and Tobago.

### Public health relevance

Wahner-Roedler et al. [27] reported 67% of physicians agreed that some CAM therapies hold promise for the treatment of symptoms, conditions, and diseases. However, the majority (70%) of physicians in the US feel that the current practice of CAM represents a threat to public health [27]. CAM usage is based largely on perceived benefits, which have given hope to many patients to accomplish wellness and improved quality of life, but little thought is spent on the multitude of interactions that may result. Furthermore, many CAM therapies lack a scientific basis and are of questionable safety and efficacy, which may lead to major health consequences: delayed treatment, disease complications, and even death. In addition, there is the possibility of herbal toxicity and herb–herb and herb–drug interactions. Oncolytic drugs have a narrow therapeutic window, and CAM use increases the risk of clinically relevant herb–anticancer drug interactions. Such a relevant interaction is that of St. John’s wort with the anticancer drugs irinotecan and imatinib. It is therefore estimated that CAM–anticancer drug interactions are responsible for substantially more unexpected toxicities of chemotherapeutic drugs and possible under-treatment of cancer patients [28]. CAM use may therefore pose a major public health problem. These concerns were raised by the WHO Traditional Medicine Strategy of 2002–2005, which emphasised four public health areas of CAM: policy; safety, efficacy, and quality; access; and rational use [2].

## Methods

### Study design and population

This cross-sectional study was conducted among all cancer patients undergoing treatment at the South West Regional Health Authority (SWRHA) of Trinidad and Tobago between March 1, 2015, and July 31, 2015. The sample size required for adequate power was 384, based on a 5% margin of error. Inclusion criteria were age > 18 years, the absence of confusion (i.e., no cognitive or behavioural problems) and communication problems, and informed consent to participate in the study. A convenience sample, comprising every sixth consecutive patient of oncology clinic attendees, and all patient attendees of the oncology treatment suite, were identified for interview. A premedical research student interviewed the consenting individuals.

### Data collection

The data collection instrument was a 37-item questionnaire covering patient demographics (age, sex, marital status, ethnicity, educational level, employment status, residence, religion, and religiosity) (8 items), oncology-related variables (5 items), and various aspects of CAM usage (types, experiences, reasons, benefits, influences, effects and consequences, source, and access to CAM) (24 items). CAM types were detailed with each type being further categorised into the many areas of practice as follows: Medicinal herbs/Biological-based medicine (Aloe Vera, Evening Primrose, Calcium, Ginger, Vitamins, etc.), Spiritual therapy/Mind-body systems (Faith healing, Divinations, Meditation, Hypnotherapy, etc.), Alternative systems (Chinese medicine, Indian/Ayurveda medicine, Acupuncture, Homeopathy), Physical therapy/Body manipulations (Chiropractic, Osteopathy, Massage, Manual healing), Energy therapies (bio-electro magnetics, Oxygen/Ozone treatment), Local/Folk remedies (Bloodletting cupping, Local surgery/Sacrification, Ritual sacrifice, Urine therapy, etc.). This questionnaire was tested and used in a previous study of cardiac patients in Trinidad [15]. Face-to-face interviews were conducted on site at clinic locations and the oncology suite. Data were collected and entered on a computer with secured access to the researcher, statistician, and research assistant.

### Statistical analysis

Statistical analysis using descriptive and inferential methods was performed using SPSS version 20 software [29]. Graphs were produced using EXCEL software after obtaining the relevant information from the SPSS output. Descriptive methods were used to obtain frequency tables and graphs. Inferential methods included tests of equality of proportions, chi-squared tests of association (e.g., Fisher’s exact test and McNemar’s test of paired proportions, as applicable) between selected socio-demographic or other attribute variables and CAM use. Binary logistic regression was used to identify predictors of CAM use. All hypotheses were tested at the 5% level of significance.

## Results

### Prevalence and patterns of CAM usage

**Fig. 1:**
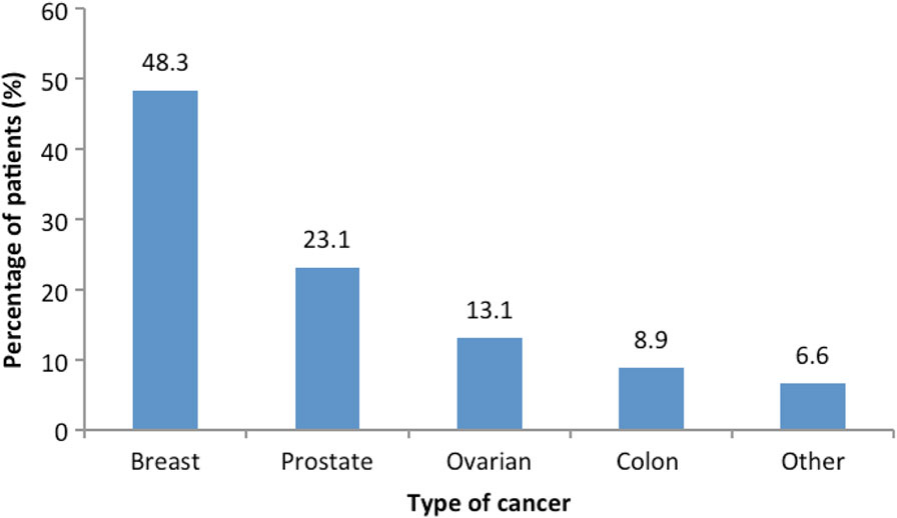
Distribution of cancer types among the study patients (*n* = 350)

Of the 384 eligible patients in the study, 350 (91.1%) completed the interview. The other 8.9% did not wish to take part in the study. The reliability of the questionnaire (Cronbach’s alpha) was 0.922. Patients were predominantly female (*n* = 249, 71.1%), Indo-Trinidadian (*n* = 210, 60.0%), secondary school educated (*n* = 152, 43.4%), and Christian (*n* = 206, 59.8%) (Table 1). CAM users and non-users had similar socio-demographic characteristics, except for the percentages of Afro-Trinidadians and of patients aged 51–60 years, which were lower among CAM users than non-users (25.5% vs. 31.9%; *p* = 0.035 and 8.0% vs. 17.8%; *p* = 0.011, respectively) (Table 1). The most common type of cancer was breast cancer (*n* = 169, 48.3%; or 67.9% of all female patients), followed by prostate cancer (*n* = 81, 23.1%; or 80.2% of all male patients) (Fig. 1). Only 2 (0.6%) patients had lung cancer. Twenty-three (6.6%) cases had other types of cancer, namely Hodgkin’s lymphoma, or bone, cervix, lung, stomach, throat, brain, ovary, liver, or throat cancer. The total number of cases did not add up to 350 because some patients had multiple cancers (e.g., bone and prostate cancer [1 patient], bone and lung cancer [1 patient], and lung, bone, and ovarian cancer [1 patient]).

**Table 1:** Socio-demographic characteristics of CAM users and non-users (*n* = 350)

Characteristic	CAM users (%) (*n* = 137)	CAM non-users (%) (*n* = 213)	*P*	Total (n)	%
Gender					
Male	43 (31.4)	58 (27.2)	0.401	101	28.9
Female	94 (68.6)	155 (72.8)	0.401	249	71.1
Age					
< 20	0 (0.0)	2 (0.9)	0.522	2	0.6
21–30	4 (2.9)	4 (1.9)	0.716	8	2.3
31–40	22 (16.1)	26 (12.2)	0.341	48	13.7
41–50	51 (37.2)	58 (27.2)	0.058	109	31.1
51–60	11 (8.0)	38 (17.8)	0.011	49	14.0
> 60	49 (35.8)	85 (39.9)	0.499	134	38.3
Religion					
Christianity	71 (51.8)	135 (63.4)	0.081	206	59.8
Hinduism	49 (35.8)	58 (27.2)	0.880	107	29.7
Islam	11 (8.0)	15 (7.0)	0.139	26	7.4
Other	6 (4.4)	5 (2.3)	0.823	11	3.1
Ethnicity					
Afro-Trinidadian	35 (25.5)	68 (31.9)	0.035	103	29.4
Indo-Trinidadian	87 (63.5)	123 (57.7)	0.097	210	60.0
Mixed	10 (7.3)	19 (8. 9)	0.835	29	8.3
Other	5 (3.6)	3 (1.4)	0.351	8	2.3
Employment status					
Unemployed	91 (66.4)	153 (71.8)	0.287	244	69.7
Employed	46 (33.6)	60 (28.2)	0.287	106	30.3
Education level					
Less than primary school	2 (1.5)	10 (4.7)	0.137	12	3.4
Primary school	44 (32.1)	83 (39.0)	0.211	127	36.3
Secondary school	63 (46.0)	89 (41.8)	0.442	152	43.4
Tertiary	28 (20.4)	31 (14.6)	0.188	59	16.9

Data are the number (percentage)

**Fig. 2:**
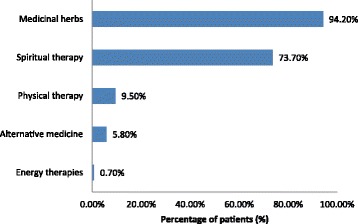
Types of complementary and alternative medicine (CAM) therapy used by cancer patients (*n* = 137)

One hundred and thirty-seven (39.1%) patients used at least one type of CAM. This figure comprised 67/169 (39.6%) breast cancer patients, 38/81 (44.4%) prostate cancer patients, 17/46 (37.0%) ovarian cancer patients, 12/31 (38.7%) colon cancer patients, and 5/23 (21.7%) patients with other types of cancer. Medicinal herbs (*n* = 129, 94.2%) and spiritual therapy (*n* = 101, 73.7%) were the most common types of CAM used (Fig. 2), regardless of cancer type (Table 2). Alternative systems, physical therapies/body manipulations, and energy therapies were not common among cancer patients.

**Table 2:** CAM use by type of cancer

CAM used	Type of cancer: Number of users (%)					
	Breast (*n* = 67)	Prostate (*n* = 36)	Ovarian (*n* = 17)	Colon (*n* = 12)	Other (*n* = 5)	*p*-value
Medicinal herbs	65 (97.0)	34 (94.4)	15 (88.2)	10 (83.36)	5 (100.0)	0.292
Spiritual therapy	46 (68.7)	27 (75.0)	13 (76.5)	10 (83.3)	5 (100.0)	0.503
Alternative medicine	4 (6.0)	5 (13.9)	1 (5.9)	3 (25.0)	0 (0.0)	0.205
Physical therapy	3 (4.5)	2 (5.6)	0 (0.0)	3 (25.0)	0 (0.0)	0.048
Energy therapy	1 (1.5)	0 (0.0)	0 (0.0)	0 (0.0)	0 (0.0)	0.902

*CAM* complementary and alternative medicine

Data are the number (percentage)

The responses to the initiation and substitution of CAM, as well as the abandonment of CM, varied among CAM users. With respect to initiation, most patients (*n* = 113, 82.5%) began using CAM while they were being treated with CM, while the remaining patients (*n* = 24, 17.5%) had begun CAM prior to CM. Most patients, 131 (95.6%) stated that they never resorted to CAM as a substitute for CM on a permanent basis. Abandonment of CM was not so common among patients since the vast majority (93.4%) were not prepared to give up CM. Only nine (6.6%) patients reported having abandoned CM for CAM at some time.

Age was the only socio-demographic variable associated with CAM use (χ^2^ = 11.365; *df* = 5; *p* = 0.045) with increasing age associated with increased use of CAM. However, neither age nor any of the other socio-demographic variables (age, sex, marital status, ethnicity, highest level of education and employment status, income, and religion) were useful predictors of CAM use.

The majority of patients (73.1% of breast cancer patients, 88.9% of prostate cancer patients, 82.4% of ovary cancer patients, 83.3% of colon cancer patients, 80.0% of patients with other types of cancer) claimed to have received some particular benefit from CAM use. The main benefits were that it would treat the condition directly (*n* = 77, 56.2%), that it would improve their psychological/emotional well-being (*n* = 68, 49.6%), that it would allow them to relax/sleep (*n* = 61, 44.5%), and that it would relieve the side-effects of conventional medicine (*n* = 36, 26.5%). Less than 10 patients failed to mention any particular item from a long, open-ended list of benefits. There was no association between the type of cancer and whether or not users claimed to have obtained particular benefits from CAM use (*p* = 0.432).

The most commonly reported reason for deciding to use CAM was the desire to try anything that could help (*n* = 96, 67.6%), followed by being congruent with their beliefs and their inner self (*n* = 81, 59.1%) (Table 3). The least common reason was that CM was too mechanistic and lacked the human touch (*n* = 12, 8.8%). Fifty-four (39.4%) patients said that they decided to use CAM because CM was too expensive.

**Table 3:** Reasons for deciding to use CAM

Reason	Type of cancer: Number of patients (%)					
	Breast (*n* = 67)	Prostate (*n* = 36)	Ovarian (*n* = 17)	Colon (*n* = 12)	Other (*n* = 5)	Total (*n* = 137)
The patient was disappointed with CM	11 (16.4)	5 (13.9)	5 (29.4)	3 (25.0)	2 (40.0)	26 (19.0)
CM was too toxic or damaging	4 (6.2)	4 (11.1)	4 (11.8)	2 (16.7)	1 (20.0)	13 (9.6)
CAM was more in keeping with personal beliefs and the inner self	38 (56.7)	22 (61.1)	10 (58.8)	9 (75.0)	2 (40.0)	81 (59.1)
The patient felt the desire to take control of treatment	20 (29.9)	14 (38.9)	6 (35.3)	1 (8.3)	1 (20.0)	42 (30.7)
CM was too mechanistic and lacked the human touch	4 (6.0)	4 (11.1)	2 (11.8)	2 (16.7)	0 (0.0)	12 (8.8)
The patient felt the desire to try everything that could help	45 (67.2)	26 (72.2)	11 (64.7)	9 (75.0)	1 (25.0)	92 (67.6)
CM was too expensive	32 (34.3)	18 (50.0)	9 (52.9)	4 (33.3)	0 (0.0)	54 (39.4)

*CAM* complementary and alternative medicine, *CM* conventional medicine. Data are the number (percentage)

### Outcome of CAM treatments

**Fig. 3:**
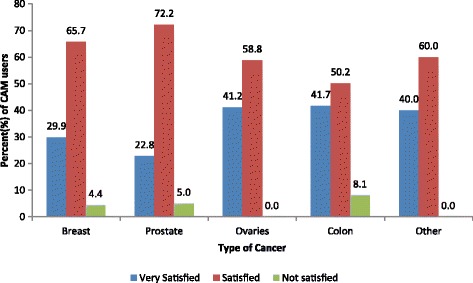
Level of satisfaction with complementary and alternative medicine (CAM) use by type of cancer

Overall, 60.0% of patients were “*Satisfied*”, 33.6% were “*Very Satisfied*”, and only 4.4% were “*Dissatisfied*”. The percentage of patients very satisfied, satisfied, and not satisfied with CAM for each type of cancer is shown in Fig. 3. Patients with prostate and breast cancer were the two groups with the highest percentages of “*Satisfied-to-Very satisfied*” patients. However, the level of satisfaction was found to be independent of cancer type. The majority of CAM users (*n* = 128, 89.8%) considered CAM helpful, and 14 (10.2%) said that it was not helpful. Of those who found CAM helpful, 94 (76.4%) had been using it once per day; 16 (13.0%), 4–6 times per week; and 12 (9.8%), 1–3 times per week. The majority of CAM users (*n* = 131, 95.6%) described the outcome of CAM treatments as good; and only 1 (0.7%) patient reported complications related to the use of CAM.

### Awareness about CAM

Patient awareness/information about CAM usage was obtained from friends (*n* = 95, 69.3%), family members (*n* = 95, 69.3%), and other patients (*n* = 60, 43.8%) (Table 4). Health personnel outside of the hospital setting were the least influential factor (*n* = 2, 1.5%). All but 3 (2.2%) patients agreed that the greater the level of knowledge a person has regarding CAM, the greater the likelihood that he/she would use it. The majority of patients (*n* = 125, 91.2%) agreed that if they had had more knowledge about CAM, they would have encouraged others to use it. Reported sources of CAM information included friends (*n* = 28; 20.4%); relatives (*n* = 21; 15.3%); a CAM practitioner (*n* = 90; 6.6%); religious groups (*n* = 8; 5.8%); and other unspecified groups or individuals (*n* = 71; 51.8%). Only 13 (9.5%) of the patients had a trained healthcare provider (allopathic or CAM practitioner) supervise or guide their CAM treatment. Furthermore, only 29 (21.2%) patients informed their physician of their use of CAM.

**Table 4:** Reported sources of CAM awareness/information

Source of CAM awareness/information	Type of cancer: Number of patients (%)					
	Breast (*n* = 67)	Prostate (*n* = 36)	Ovarian (*n* = 17)	Colon (*n* = 12)	Other (*n* = 5)	Total (*n* = 137)
Health personnel outside the hospital	1 (1.5)	1 (2.8)	0 (0.0)	0 (0.0)	0 (0.0%)	2 (1.5%)
In-hospital health personnel	14 (20.9)	5 (13.9)	1 (5.9)	3 (25.0)	1 (20.0)	24 (17.5)
Friends	46 (68.7)	30 (83.3)	9 (52.9)	8 (66.7)	2 (40.0)	95 (69.3)
Family members	46 (68.7)	29 (80.6)	8 (47.1)	9 (75.0)	3 (60.0)	95 (69.3)
CAM practitioner	4 (6.0)	5 (13.9)	0 (0.0)	3 (25.0)	0 (0.0)	12 (8.8)
Mass media	9 (11.9)	2 (5.6)	8 (47.1)	1 (8.3)	1 (20.0)	20 (14.6)
Religious groups	15 (22.4)	14 (38.9)	5 (29.4)	2 (16.73)	0 (0.0)	36 (26.3)
Other patients	29 (43.3)	17 (42.7)	6 (35.3)	8 (66.7)	0 (0.0)	60 (43.8)
Other persons (unspecified)	1 (1.5)	1 (2.8)	2 (11.8)	0 (0.0)	0 (0.0)	4 (2.9)

*CAM* complementary and alternative medicine. Data are the number (percentage)

## Discussion

In this study, the overall prevalence of CAM among cancer patients was 39.1%. In this study, the prevalence of CAM varied for different cancers, namely ovarian (37.0%), colon (38.7%), breast (39.6%), and prostate cancer (44.4%). This was in agreement with a previous study [21]. Of all socio-demographic variables tested, CAM usage was only associated with age. However, neither age nor any of the other socio-demographic variables were useful predictors for CAM usage.

Herbs were the most common type of CAM used (94.2%), followed by spiritual therapy (73.7%). Molassiotis et al. also reported herbs as the top CAM therapy among cancer patients in 9 out of 14 countries, including Turkey, Israel, Serbia, Czech Republic, Denmark, Italy, Switzerland, Spain, and Greece [21]. The majority of CAM users use at least 1 herb. Nonetheless, patients were not prepared to give up CM (93.4%). This may reflect patients’ lack of complete trust in CAM and fear of losing the benefits of CM. CAM was used mainly because of a desire to try anything (67.6%), followed by being congruent with their beliefs and inner self (59.1%).

CAM is used in nearly all types of cancer. There were no significant differences in the choice of medicinal herbs and spiritual CAM therapies, which were the most commonly used types of CAM among all types of cancer. However, while patients recognise the perceived value of CAM, 78.8% of patients from this study failed to communicate their use of CAM to their health care provider. In contrast, Saxe et al. [30] found that there was a high disclosure rate of CAM to physicians, with as much as 85% of breast cancer patients disclosing their use of naturopathy to their physicians [30]. This contrasts with the findings of Eisenberg et al. [31], who found that 63%–72% of patients practiced CAM without informing their health care providers. Non-disclosure may not be in the best interest in patient care, since vital information required for the management of patients is lost. Furthermore, few doctors are prepared to enquire from patients about CAM usage [32]. Patients also fear disclosing CAM information. This has led the US National Center for Complementary and Alternative Medicine (NCCAM) to launch the “Time to Talk” campaign encouraging both health care providers and patients to communicate about CAM use [33].

The majority of patients seem to have no clear guidance or basis for appropriate CAM use, with only 13 (9.5%) patients having their CAM supervised or guided by a trained health care provider. Furthermore, only a small percentage of patients (6.6%) stopped using CM to use CAM. According to van Kleffens and van Leeuwen [11], patients refused CM treatment because of the desire to stay in control, fear of losing breasts, or not wanting to fight any more. In contrast, a 2013 prospective study in terminally ill cancer patients found that CAM did not provide any definite survival benefit or improved health-related quality of life [34]. More unfavourable findings regarding CAM usage was revealed in a 2003 study on cancer patients from Norway, which revealed higher death rates (79%) among CAM users than non-users (65%) [35]. CAM usage is attributed to the increasing demand and expectations for more holistic and comprehensive care [36]. It is perceived as being “natural” and “safe” [37], effective [38], and with fewer adverse effects [39]. CAM users experience a feeling of being in control, coping, and adjustment. The rationale behind CAM, whether it be problem-focused (strategies based on biological therapies) or emotion-focused (based on prayers and meditation techniques), is reported to be largely based on erroneous logic and science [40]. However, some studies find that the benefits of CAM should not be disregarded, and oncologists should familiarize themselves with commonly used CAM, in order to provide their patients with proper guidance in all aspects of their treatment [41].

### Public health relevance

High satisfaction levels have overshadowed or downplayed the complications of herbal toxicity, herb–herb and herb–drug interactions; or treatment problems because of CAM usage or CM avoidance. Furthermore, its use continues because of the lack of regulations in Trinidad and Tobago [42], exposure and influences from a multitude of sources, and increasing availability of various types of CAM. The lack of professional CAM supervision, the growing number of CAM users, low disclosure rates, lack of meaningful advice from CM providers, and poor monitoring contribute to a major public health problem. The simultaneous practice of CAM and CM necessitates greater understanding, communication, and integration of these 2 forms of treatment.

### Limitations

This study was conducted in a single public health institution. The sample was skewed towards a less economically privileged population. Because of the sample size, subpopulation analysis would be difficult since there would be insufficient power to draw meaningful conclusions. Patients may withhold information that they may be embarrassed about. The database is based entirely on the memory and truthfulness of patients’ responses, which may have involved bias. The results and conclusions are unique to our setting in Trinidad and generalisations would be difficult unless the populations are similar.

## Conclusions

The prevalence of CAM use was relatively high (39.1%) among cancer patients. The frequent use of herbs (93.4%) and spiritual therapy (73.7%), the abandonment of CM for CAM (although only 6.6%), the failure by the vast majority (78.8%) of CAM users to inform their health care provider, and the high satisfaction (over 90%) with CAM use may have major health implications (delayed treatment, drug interactions, disease complications, and the possibility of death). Females, Indo-Trinidadians, and patients in the age range of 41–50 years are the main users. Patients’ use of CAM was mainly to try anything that might help, and to remain congruent with their beliefs and inner self. Other reasons included wellbeing, relaxation, counteracting the side-effects of CM, and cost. Patients were influenced to use, or introduced to CAM, by friends, followed by family, other patients, religious groups, mass media, in-hospital personnel, CAM practitioners, and health personnel outside of a hospital. Patients believe more education would encourage them to use more CAM.

## Abbreviations

CAM: Complementary and alternative medicine
CM: Conventional medicine
HCP: Health care provider
NCCAM: National Center for Complementary and Alternative Medicine
NHS: National Health Service
SFGH: San Fernando General Hospital
SWRHA: South West Regional Health Authority
WHO: World Health Organization
